# Cholinergic Nociceptive Mechanisms in Rat Meninges and Trigeminal Ganglia: Potential Implications for Migraine Pain

**DOI:** 10.3389/fneur.2017.00163

**Published:** 2017-04-27

**Authors:** Irina Shelukhina, Nikita Mikhailov, Polina Abushik, Leniz Nurullin, Evgeny E. Nikolsky, Rashid Giniatullin

**Affiliations:** ^1^Department of Neurobiology, A. I. Virtanen Institute for Molecular Sciences, University of Eastern Finland, Kuopio, Finland; ^2^Shemyakin-Ovchinnikov Institute of Bioorganic Chemistry, Russian Academy of Sciences, Moscow, Russian Federation; ^3^Laboratory of Biophysics of Synaptic Processes, Kazan Institute of Biochemistry and Biophysics, Kazan, Russian Federation; ^4^Open Laboratory of Neuropharmacology, Kazan Federal University, Kazan, Russian Federation; ^5^Laboratory of Neurobiology, Kazan Federal University, Kazan, Russian Federation

**Keywords:** migraine, acetylcholine, nicotine, acetylcholine receptor, meninges, sensory neurons, mast cells

## Abstract

**Background:**

Parasympathetic innervation of meninges and ability of carbachol, acetylcholine (ACh) receptor (AChR) agonist, to induce headaches suggests contribution of cholinergic mechanisms to primary headaches. However, neurochemical mechanisms of cholinergic regulation of peripheral nociception in meninges, origin place for headache, are almost unknown.

**Methods:**

Using electrophysiology, calcium imaging, immunohistochemistry, and staining of meningeal mast cells, we studied effects of cholinergic agents on peripheral nociception in rat hemiskulls and isolated trigeminal neurons.

**Results:**

Both ACh and carbachol significantly increased nociceptive firing in peripheral terminals of meningeal trigeminal nerves recorded by local suction electrode. Strong nociceptive firing was also induced by nicotine, implying essential role of nicotinic AChRs in control of excitability of trigeminal nerve endings. Nociceptive firing induced by carbachol was reduced by muscarinic antagonist atropine, whereas the action of nicotine was prevented by the nicotinic blocker d-tubocurarine but was insensitive to the TRPA1 antagonist HC-300033. Carbachol but not nicotine induced massive degranulation of meningeal mast cells known to release multiple pro-nociceptive mediators. Enzymes terminating ACh action, acetylcholinesterase (AChE) and butyrylcholinesterase, were revealed in perivascular meningeal nerves. The inhibitor of AChE neostigmine did not change the firing *per se* but induced nociceptive activity, sensitive to d-tubocurarine, after pretreatment of meninges with the migraine mediator CGRP. This observation suggested the pro-nociceptive action of endogenous ACh in meninges. Both nicotine and carbachol induced intracellular Ca^2+^ transients in trigeminal neurons partially overlapping with expression of capsaicin-sensitive TRPV1 receptors.

**Conclusion:**

Trigeminal nerve terminals in meninges, as well as dural mast cells and trigeminal ganglion neurons express a repertoire of pro-nociceptive nicotinic and muscarinic AChRs, which could be activated by the ACh released from parasympathetic nerves. These receptors represent a potential target for novel therapeutic interventions in trigeminal pain and probably in migraine.

## Introduction

In migraine pathophysiology, much attention was traditionally paid to the role of trigeminal innervation of meninges, likely representing a triggering zone for migraine pain (1–3). Peripheral primary afferent excitation is followed by activation of neurons in the trigeminocervical complex (4).

In contrast to trigeminal system, considerably less is known about the functional role of parasympathetic innervation of these tissues. Nevertheless, it has been shown that parasympathetic fibers originating from the parasympathetic sphenopalatine ganglion (SPG) can innervate and interact, *via* released acetylcholine (ACh), with somatic trigeminal nerves located around essential meningeal vessels (5). Moreover, parasympathetic nociceptive traffic can participate in the reflex connecting the trigeminal nucleus caudalis and SPG (central trigeminal–parasympathetic reflex) to control the meningeal blood flow (6, 7).

Electrical stimulation of the SPG can induce one of migraine-related effects, such as vasodilation and plasma protein extravasation in the dura mater likely mediated by muscarinic ACh receptors (mAChRs) (8). In contrast, the blockade of SPG can reduce migraine features in patients (9). It is also known that carbachol, a typical agonist of ACh receptors (AChRs), can induce headaches, but not migraine-like attacks, in patients with migraine without aura (10). This ACh analog is an effective vasodilator of cranial vessels (11). Moreover, it has been shown (12) that injection of botulinum neurotoxin/A (Botox), which inhibits the exocytosis of ACh, can reduce plasma protein extravasation, a phenomenon often associated with migraine. Interestingly, smokers more often suffer from migraine than non-smokers (13), suggesting a potential pro-nociceptive action of nicotine. Whereas nicotinic ACh receptors (nAChRs) are likely involved in pathophysiology of neuropathic pain (14), the anti-nociceptive rather than pro-nociceptive action of nicotine has been reported in *in vivo* pain models (15–17). One reason for such discrepancies could be the presence of various molecular targets along sensory pathways for nicotine, which apart from ligand-gated nAChRs can also activate pro-nociceptive TRPA1 receptors (18, 19).

It is widely accepted nowadays that the key role in migraine pathology belongs to the neuropeptide CGRP, which is released in venous blood flow during migraine attack (20). Carbachol did not change the release of CGRP from meninges; rather, it produced a depressant effect after the sensitization of meninges with inflammatory mediators (5). Carbachol also released histamine from isolated dura mater (21) suggesting degranulation of local mast cells, which are widely presented in meninges and likely play an important role in migraine pathology (22).

In the current project, using *ex vivo* rat hemiskull preparation with preserved innervation, we showed that the main AChR agonists, ACh, carbachol, and nicotine, all activated nociceptive firing in meningeal nerves and that carbachol (but not nicotine) induced degranulation of dural mast cells. Ca^2+^ imaging allowed us to confirm the pro-nociceptive action of nicotine and carbachol at the level of single trigeminal neurons.

## Materials and Methods

### Animals

Wistar rats of both sexes from the Animal House of the University of Eastern Finland were used in this study. P9–P13 rats were used for trigeminal cell culture preparation and mast cell degranulation assay, whereas adult (P35–P36) rats were used for electrophysiology and immunohistochemistry.

### Electrophysiology

Preparation of male rat (P35–P36) hemiskulls was performed as published earlier (23, 24). Briefly, after decapitation, the skin and muscles were removed from the skull, which was dissected mid-sagitally, and both brain hemispheres were gently removed leaving dura mater untouched with preserved trigeminal innervations. A hemiskull was placed into a recording chamber and was continuously superfused with oxygenated (5% CO_2_/95% O_2_) artificial cerebrospinal fluid (ACSF) containing (in millimolars): NaCl 120, KCl 3, CaCl_2_ 2, MgCl_2_ 1, NaH_2_PO_4_ 1, NaHCO_3_ 25, and glucose 10, pH 7.3 (6–7 ml/min). The nervus spinosus, which is a part of the mandibular branch of the trigeminal nerve (25) was exposed by a careful dissection of dura mater and thereafter was placed into a fire-polished recording glass microelectrode filled with ACSF. Investigated substances were applied to the recording chamber for 10–20 min *via* superfusion. Low-noise digital amplifier (ISO 80, WPI Inc., Sarasota, FL, USA) was used for recordings. Signals were visualized and analyzed with the WinEDR V3.4.6 software (Strathclyde University, Glasgow, UK). Spontaneous and drug-induced action potentials generated in the distal parts of the transected nervus spinosus were recorded at room temperature. Spike frequencies (number of spikes per every 2 min) were estimated and analyzed using OriginPro 9.1 software (Microcal, Northampton, MA, USA). For control values, the frequency of spikes during 10 min prior to drug application was taken.

### Immunostaining of Acetylcholinesterase (AChE) and Butyrylcholinesterase (BuChE)

Isolated male rat dura mater preparations were fixed during 60 min in 2% p-formaldehyde (Sigma-Aldrich, USA) and rinsed three times for 30 min in the phosphate buffer (PBS, Sigma-Aldrich). Then, the preparations were consecutively incubated for 30 min in 0.3% Triton X-100 (Sigma-Aldrich) for 15 min in a solution containing 5% goat serum (Sigma-Aldrich), 1% bovine serum albumin (BSA, Sigma-Aldrich), and 0.3% Triton X-100 and for 15 min in a solution of 1% BSA and 0.3% Triton X-100 (solution A). These solutions were based on PBS.

Next, preparations were incubated for 15 h at 4°C in the solution A containing goat polyclonal antibody to AChE (dilution 1:1,000, Santa Cruz Biotechnologies, USA), or mouse monoclonal antibody to BuChE (dilution 1:1,000) and rabbit polyclonal antibodies to the neurofilament NF-H (1:1,000, Santa Cruz Biotechnologies, USA). The preparations were rinsed three times in the solution A and then incubated for 1 h at room temperature with the appropriate secondary antibodies conjugated to Alexa Fluor 488 or 647 (Invitrogen, USA) diluted 1:1,000 in the solution A.

After washing in PBS, the preparations were exposed to PBS solution diluted by glycerol (1:1) and used for microscopy (63×, oil immersion, NA = 1.4) with a laser scanning confocal microscope Zeiss LSM 510 Meta (Carl Zeiss, Germany).

### Dural Mast Cell Degranulation

To study the action of cholinergic drugs on degranulation of dural mast cells, hemiskulls of Wistar rats of both sexes were used. One control hemiskull was filled for 20 min with the basic saline solution (BSS), containing (in millimolars) 152 NaCl, 5 KCl, 10 HEPES, 10 glucose, 2.6 CaCl_2_, 2.1 MgCl_2_ (pH adjusted to 7.4 with NaOH), whereas others were filled with BSS containing 50 µM carbachol or 100 µM nicotine. Then, the hemiskulls were left in 4% PFA solution for at least 4 h. After that meninges were dissected carefully and placed on a microscope slide, where they were stained with 0.1% toluidine blue (26). Images of meninges were acquired with Olympus AX70 microscope (20× objective). Homogeneously stained and well-shaped mast cells were classified as non-degranulated, whereas pale poorly stained mast cells as well as mast cells with distorted borders were classified as degranulated (22, 26). To study an effect of carbachol and nicotine on mast cells degranulation, 10 randomly chosen, same in different animals, perivascular areas of meninges enriched by mast cells were analyzed (27). Then, total number and number of degranulated mast cells were counted in a blind manner.

### Primary Culture of Rat Trigeminal Sensory Neurons

Trigeminal ganglion (TG) cell cultures were prepared as described previously (28). Briefly, trigeminal ganglia of male rats were isolated, and cells were enzymatically dissociated by trypsin (0.5 mg/ml, Sigma-Aldrich), collagenase (1 mg/ml, Sigma-Aldrich), and DNAse (0.2 mg/ml, Sigma-Aldrich) for 20 min. Isolated cells were plated on coverslips coated with poly-l-lysine (0.2 mg/ml, Sigma-Aldrich, MO, USA) and were cultured in F12 medium (Gibco, USA) at 37°C, 5% CO_2_ for 48 h prior to an experimental treatment.

### Calcium Imaging of Trigeminal Neurons

Primary TG cells kept in culture for 2 days were rinsed with basic solution (BS) containing (in millimolar) 152 NaCl, 2.5 KCl, 10 HEPES, 10 glucose, 2 CaCl_2_, 1 MgCl_2_ (pH adjusted to 7.4) followed by loading with 2 µM fluo-3 acetoxymethyl ester (Invitrogen, USA) at 37°C for 45 min in BS. After 20 min postincubation, dishes were transferred to TILL Photonics imaging system (TILL Photonics GmbH, Munich, Germany) and were constantly perfused (at 1.2 ml/min) with BS. The setup was equipped by fast perfusion system (Rapid Solution Changer RSC-200, BioLogic Science Instruments, Grenoble, France), which allowed rapid (exchange time ~30 ms) application of various compounds. Cells were viewed *via* Olympus IX-70 (Tokyo, Japan) microscope with a specific filter of 488 nm wavelength using 10× objective. Images were collected using CCD camera (SensiCam, PCO imaging, Kelheim, Germany) at sampling frequency set to 2 fps. Cells were further characterized by their responsiveness to a brief application of high potassium (50 mM KCl with compensated osmolarity) as a marker for neurons.

### Statistical Analysis

Nociceptive firing was statistically analyzed using OriginPro 9.1 software (Microcal, Northampton, MA, USA) and SigmaPlot 11.0 (Systat Software Inc., CA, USA). One-way repeated measures ANOVA with Tukey *post hoc* test was used. Mast cell degranulation data were analyzed using two-tailed Mann–Whitney *U* test with GraphPad Prism software (La Jolla, CA, USA). Two-tailed Student’s *t*-test [OriginPro 9.1 software (Microcal, Northampton, MA, USA)] was applied to evaluate the significance of increase in basal meningeal firing after 1 µM CGRP pre-application. Results are expressed as mean of data ± SE unless otherwise stated. In all tests, *p* < 0.05 was taken as significant.

## Results

### Nociceptive Firing Induced by Carbachol

To study if cholinergic agents can stimulate or inhibit meningeal nociceptive signaling, we applied various AChR agonists directly to the rat hemiskull preparation with preserved dura mater innervation. To this end, we recorded spontaneous nociceptive firing of meningeal trigeminal nerves in basal conditions, then the preparation was superfused with a ligand solution, and changes in spike frequency were monitored.

**Figure 1:**
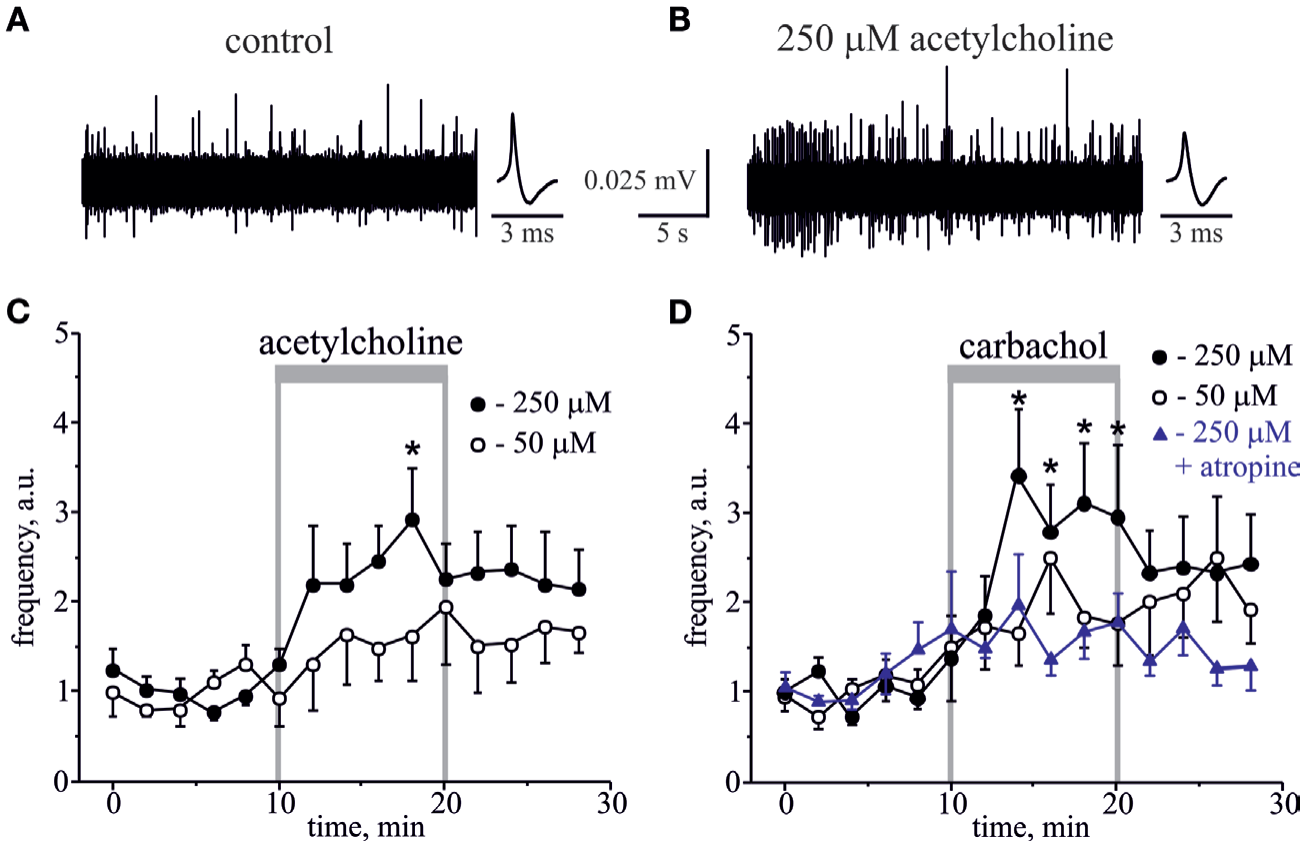
**Action of acetylcholine (ACh) and carbachol on nociceptive firing in the meningeal trigeminal nerves**. **(A)** Representative traces of trigeminal nociceptive firing and average spike shape in control conditions and **(B)** during application of 250 µM ACh. **(C)** The time-course of changes of nociceptive spike frequency during application of 50 and 250 µM ACh. **(D)** The time-course of changes of nociceptive spike frequency during application of 50 and 250 µM carbachol and 250 µM carbachol in the presence of 1 µM atropine. Each time point represents a mean spike frequency for 2 min of recording (mean ± SEM, *n* = 6–10, one-way repeated measures ANOVA, Tukey test, *p < 0.05).

First, we applied the natural agonist ACh to rat hemiskull preparation. Application of 250 µM ACh significantly increased nociceptive firing (Figures 1A–C). Figures 1A,B show representative spikes in control and after ACh application, respectively. Notice that the amplitude and the shape of spikes were not significantly changed after ACh treatment (Figures 1A,B, *inset*). On average, the frequency of spikes was increased by 2.5-fold by 10 min of 250 µM ACh application (*n* = 6, *p* < 0.05, Figure 1C). Lower (50 µM) concentration of ACh slightly increased spike frequency, but this effect was not significant (*n* = 6, *p* > 0.05, Figure 1C).

Because ACh, an endogenous agonist of both mAChRs and nAChRs, quickly degrades in live tissues, we next used the stable agonist carbachol, to test whether it can induce similar or higher level of firing. Although ACh demonstrates comparable activity towards both classes of AChRs, carbachol is more potent to mAChR, to which it demonstrates nanomolar affinity in contrast to micromolar constants for its binding to nAChRs (29–32). Like low doses of ACh, 50 µM carbachol showed a clear tendency to increase firing, but this effect was not significant (*n* = 10, *p* > 0.05, Figure 1D). However, application of 250 µM carbachol to the rat hemiskull preparation significantly increased the number of nociceptive spikes peaking by 5–10 min of drug application (*n* = 6, *p* < 0.05, Figure 1D). Moreover, the significantly increased number of spikes was detected even 6 min after carbachol withdrawal. Notice that the same concentration of ACh induced the significant changes in firing only after 10 min of the compound application consistent with higher pro-nociceptive efficacy of carbachol.

The pro-nociceptive action of carbachol was largely reduced by pretreatment with broad spectrum muscarinic antagonist, atropine. Thus, in the presence of 1 µM atropine, 250 µM carbachol only slightly increased nociceptive firing, but this effect was non-significant (*n* = 7, *p* > 0.05, Figure 1D).

### Involvement of Nicotinic Receptors in Nociceptive Firing

**Figure 2:**
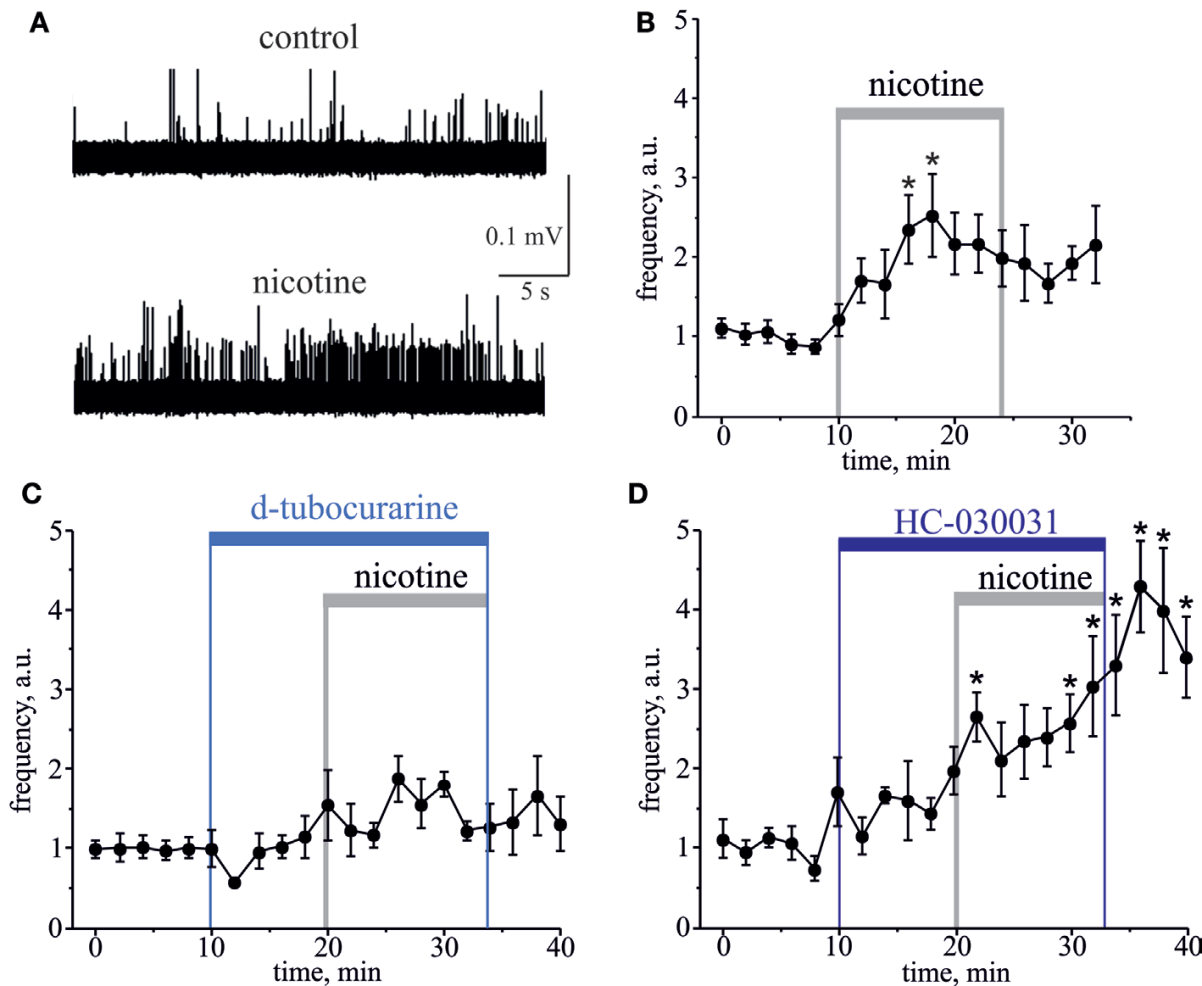
**Action of nicotine on nociceptive firing in the meningeal trigeminal nerves**. **(A)** Representative traces of trigeminal nociceptive spikes in control conditions and during application of 100 µM nicotine. **(B)** The time-course of changes of nociceptive spike frequency during application of 100 µM nicotine. **(C)** The time-course of changes of nociceptive spike frequency during application of 100 µM nicotine in the presence of 50 µM d-tubocurarine and **(D)** in the presence of 25 µM HC-030031. Notice that d-tubocurarine (a non-selective nicotinic antagonist) prevented this effect, but HC-030031 (a specific TRPA1 blocker) did not prevent the pro-nociceptive action of nicotine. Each time point represents a mean spike frequency for 2 min (mean ± SEM, *n* = 7–9, one-way repeated measures ANOVA, Tukey test, *p < 0.05).

Essential evidence suggests that ligand-gated nicotinic receptors (nAChRs) are also expressed by nociceptive sensory neurons to modulate the nociceptive signaling (33–37). During carbachol application, the role of nAChRs could be masked by simultaneous activation of multiple mAChRs often producing functionally opposite effects on nociceptive neurotransmission (38–40). Therefore, to investigate directly a contribution of nAChRs to the nociceptive signaling in meninges, we used the specific nAChRs agonist nicotine. Nicotine (100 µM) induced a strong and prolonged nociceptive firing in trigeminal nerves (Figure 2A). This effect was peaking at 8–10 min (*n* = 8, *p* < 0.05, Figure 2B) of nicotine application. Consistent with the involvement of nAChRs, pre-application of 50 µM d-tubocurarine completely prevented the pro-nociceptive effect of nicotine (*n* = 9, *p* > 0.05, Figure 2C). TRPA1 receptors have also been suggested as molecular targets sensitive to this agonist (18, 19). However, in rat meninges, TRPA1 receptors are unlikely the target for nicotine, as the selective TRPA1 blocker 25 µM HC-030031 did not prevent the pro-nociceptive action of nicotine (*n* = 7, Figure 2D).

### AChE, BuChE, and the Role of Endogenous ACh

**Figure 3:**
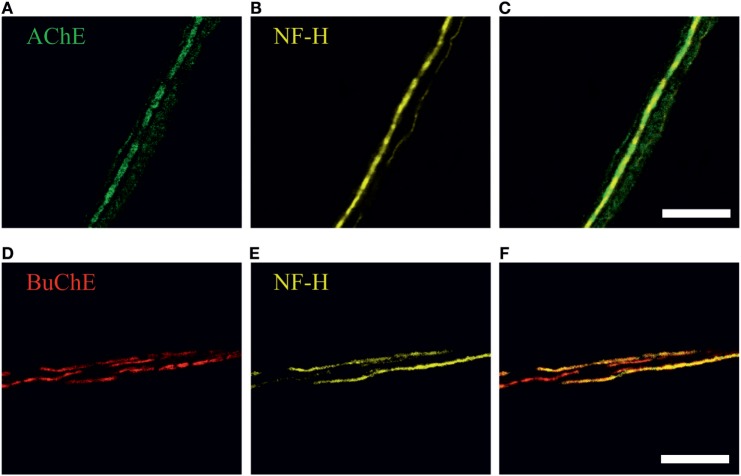
**Immunohistochemical staining of (A) acetylcholinesterase (AChE, green), (D) butyrylcholinesterase (BuChE, red) and (B,E) neurofilaments (NF-H, neurofilaments heavy chain, *yellow*) in perivascular nerve fibers in rat dura mater preparation**. Co-localization with neurofilaments, markers of myelinated axons, is shown in **(C)** and **(F)**. Scale bar, 40 µm.

To study a role of endogenous meningeal cholinergic system in rat dura mater, we explored the expression and localization of AChE and BuChE, two key enzymes implicated in cholinergic signaling by termination of ACh action. The cholinesterase-positive nerve fibers were found mainly in perivascular regions (*n* = 7 hemiskulls), although spare labeling was observed across the dura mater. Figures 3A–F present a co-expression of AChE and BuChE with neurofilaments (NF-H) in nerve fibers located nearby meningeal blood vessels. Such localization of both enzymes in dura mater suggests their role in control of ACh-mediated signaling.

**Figure 4:**
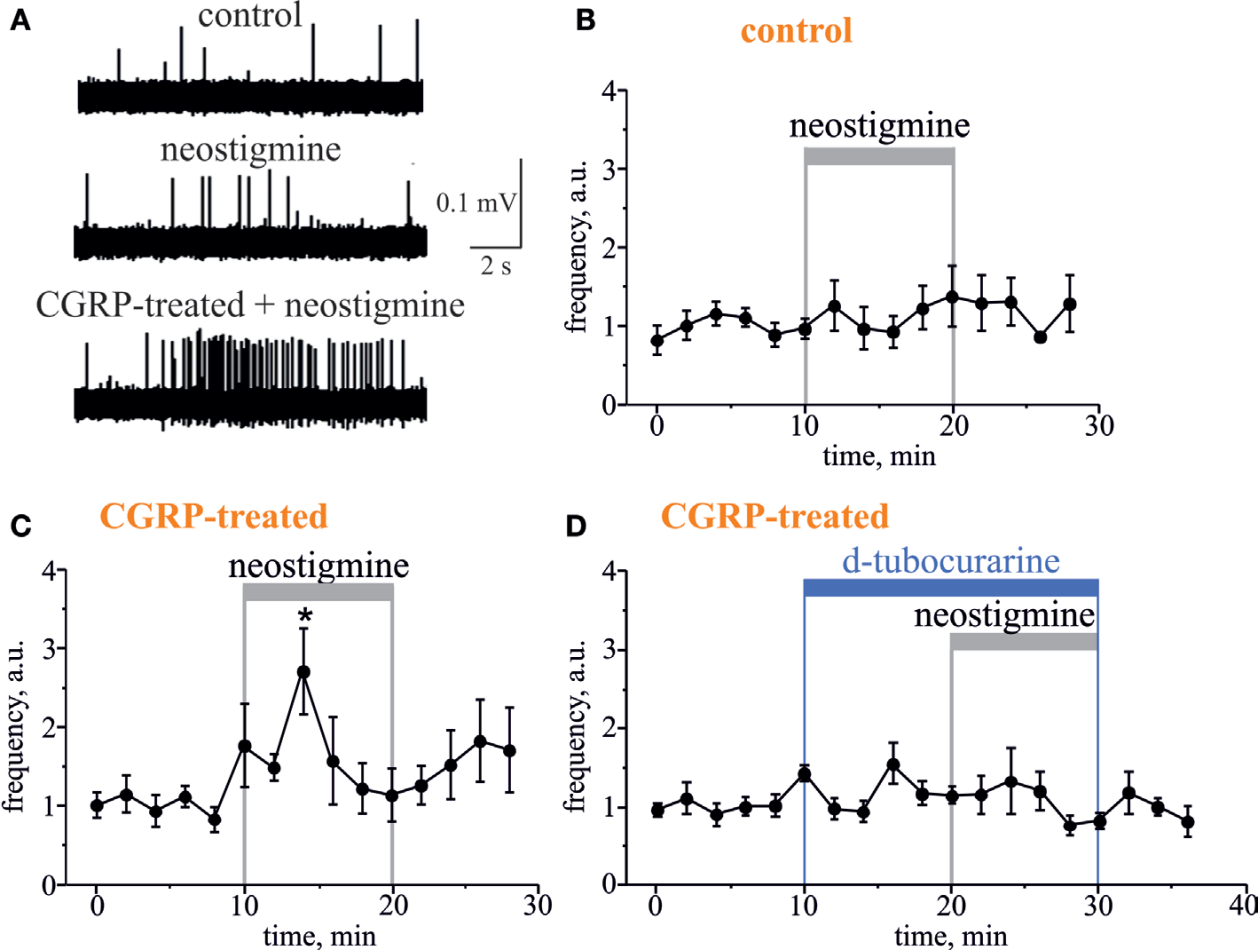
**Pro-nociceptive action of neostigmine in CGRP-sensitized hemiskull preparation**. **(A)** Representative traces of trigeminal nociceptive firing in control conditions, during 12.5 µM neostigmine application and neostigmine application in 1 µM CGRP-pretreated preparation. **(B)** The time-course of changes of nociceptive spike frequencies after application of 12.5 µM neostigmine in a control and **(C)** in CGRP-sensitized hemiskull (preincubation in 1 µM CGRP for 2 h). **(D)** The time-course of changes of nociceptive spike frequency in CGRP-sensitized hemiskull after application of 12.5 µM neostigmine in the presence of 50 µM d-tubocurarine. Notice that application of a non-selective nicotinic antagonist d-tubocurarine prevented nociceptive firing induced by neostigmine. Each plot point represents a mean spike frequency for 2 min (mean ± SEM, *n* = 5–7, one-way repeated measures ANOVA, Tukey test, *p < 0.05).

Further, to test the involvement of endogenous ACh in regulation of meningeal nociceptive firing, we applied the classical inhibitor of AChE neostigmine to the hemiskull preparation, to increase the concentration of endogenous ACh around nerve fibers. Neostigmine (12.5 µM) did not change the nociceptive firing *per se* (*n* = 5, *p* > 0.05, Figures 4A,B). However, a significant neostigmine-induced increase in nociceptive firing was observed after 2 h exposure of meninges to the migraine mediator calcitonin gene-related peptide (CGRP, 1 µM). Thus, in this case, the number of spikes was increased by 6 min action of neostigmine by 2.7-fold (*n* = 7, *p* < 0.05, Figures 4A,C). The basal level of trigeminal firing was not changed after 2 h preincubation with 1 µM CGRP (372 ± 124 spikes in 10 min recording, *n* = 12) versus 385 ± 75 spikes in control (*n* = 12, *p* > 0.05). Importantly, co-application of 50 µM d-tubocurarine (non-selective nAChR antagonist) prevented the facilitatory pro-nociceptive action of neostigmine (*n* = 5, *p* < 0.05, Figure 4D), suggesting the major role of nicotinic AChRs in mechanism of neostigmine-induced firing.

### Carbachol but Not Nicotine Degranulates Dural Mast Cell

**Figure 5:**
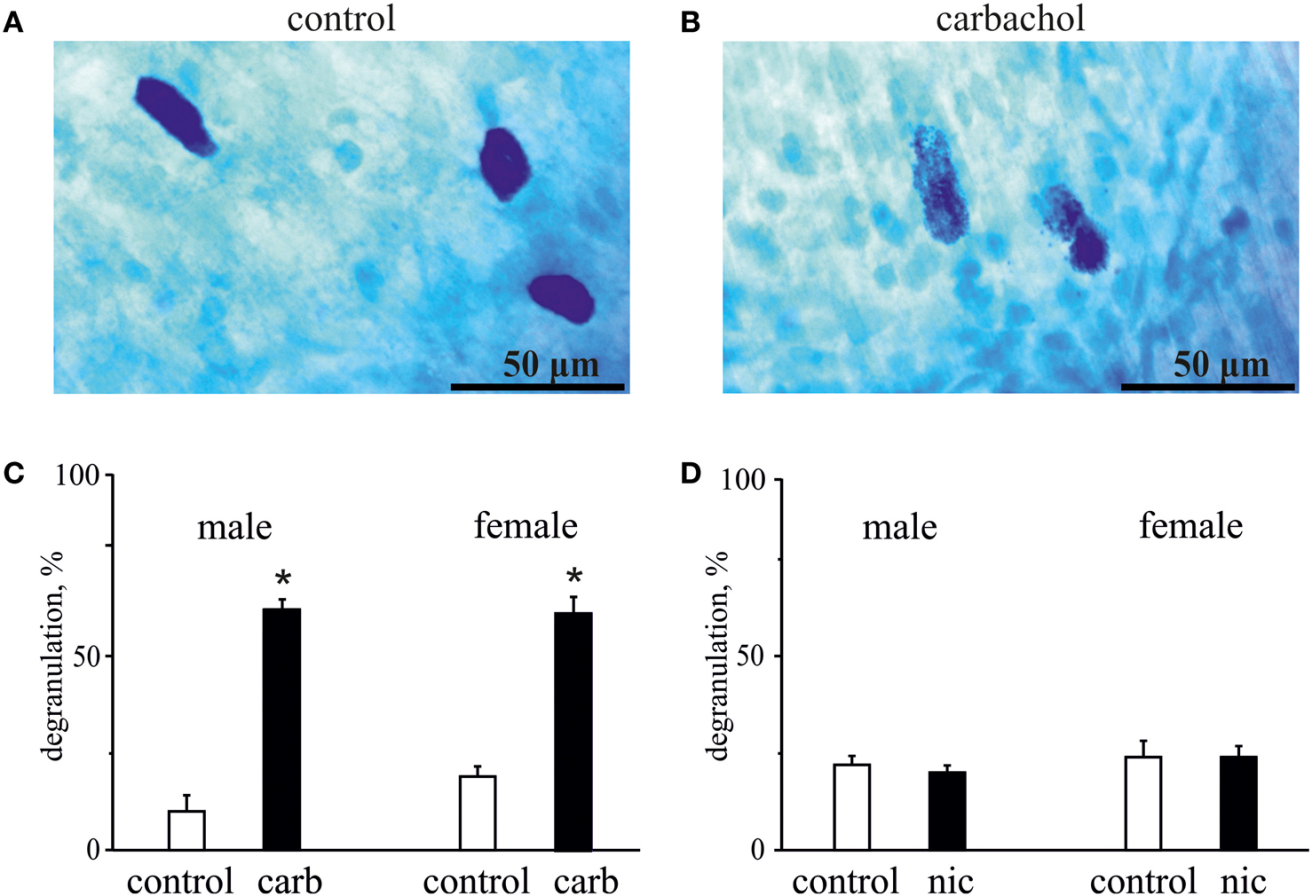
**Action of carbachol and nicotine on degranulation of mast cells**. **(A)** Example of intact well-shaped mast cells in control. **(B)** Example of degranulated mast cells (notice distorted borders) in the presence of 50 µM carbachol. **(C)** Histograms showing the effect of 50 µM carbachol on degranulation of mast cells obtained from male and female rats. **(D)** Histograms showing the effect of 100 µM nicotine on degranulation of mast cells obtained from male and female rats. Notice lack of nicotine effect on mast cell degranulation. Mean ± SD, *n* = 4, Mann–Whitney *U* test, *p < 0.05.

As degranulation of mast cells has been recently suggested as one of potential triggers of migraine (22, 41), we next tested the ability of carbachol and nicotine to induce degranulation of dural mast cells. In these experiments, we also explored a possible role of gender in the ability of these cholinergic agents to induce degranulation. Figure 5 shows representative samples of mast cells in control (Figure 5A) and after application of 50 µM carbachol (Figure 5B). Pooled data in Figure 5C show that the application of 50 µM carbachol to the whole mount meningeal preparation for 20 min induced a significant degranulation of mast cells in both sexes. Thus, in males, the level of degranulation in control (vehicle treatment) was 10 ± 8% (*n* = 4), whereas it was largely enhanced up to 62 ± 5% after 50 µM carbachol treatment (*n* = 4, *p* < 0.05). In females, there was 19 ± 5% of degranulation in control (*n* = 4), and the large enhancement of degranulation up to 61 ± 8% was observed after 50 µM carbachol exposure (*n* = 4, *p* < 0.05).

In contrast to carbachol, the concentrations of nicotine, which induced the nociceptive firing, did not promote degranulation of mast cells (Figure 5D) suggesting only a direct action of this agonist on nerve terminals. Thus, in males, the level of mast cells degranulation was 22 ± 4% in control (*n* = 4) and 20 ± 4% after application of 100 µM nicotine (*n* = 4, *p* > 0.05). Likewise, in females, these values were 24 ± 8% for vehicle (*n* = 4) and 24 ± 5% for nicotine treatment (*n* = 4, *p* > 0.05).

Thus, the pro-nociceptive action of nicotine was clearly mast cells independent, whereas the action of carbachol could potentially be, at least partly, mediated by products released from degranulated mast cells.

### Calcium Transients Induced by Nicotine in Trigeminal Neurons

The somas of TG neurons are important components of peripheral trigeminal nociceptive system, and they could be implicated in development of migraine pain (1). To study the effect of cholinergic agents in this part of the trigeminal nociceptive system, we used the live imaging technique to monitor Ca^2+^ signals in neurons isolated from TG. Application of 50 mM KCl at the end of protocol was used to identify neurons (42). To study the neurochemical profile of the neurons responding to cholinergic agonists, we tested if they were activated by the classical algogen capsaicin operating *via* TRPV1 receptors (43). We found a variable profile of trigeminal neurons as some cells responded to nicotine, some only to capsaicin and the fraction of cells responded to both agonists.

**Figure 6:**
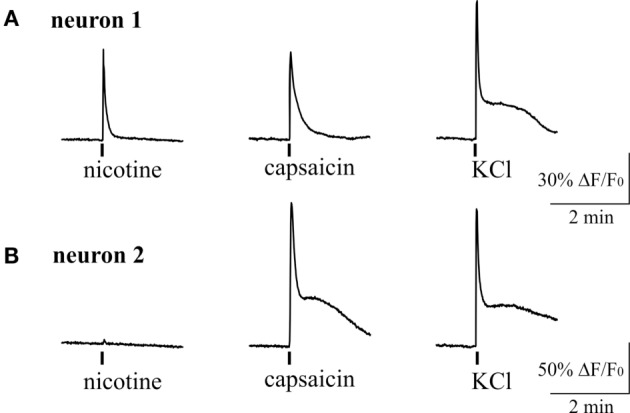
**Intracellular Ca^2+^ responses induced by nicotine and capsaicin in trigeminal neurons**. **(A)** Example of intracellular calcium transient induced by 100 µM nicotine in TRPV1-positive neuron (sensitive to 200 nM capsaicin). **(B)** Example of calcium transient activated by 200 nM capsaicin in nicotine-negative neuron. All agonists were applied for 2 s with at least 2-min intervals between applications. Application of 50 mM KCl was used as a marker for identification of neurons. Statistical analysis of these experiments is given in the Section “Results.”

Figure 6 shows example of two neurons exposed to 100 µM nicotine. In one trigeminal neuron (Figure 6A), nicotine induced a large fast Ca^2+^ transient, and later this neuron responded also to capsaicin. In the other neuron (Figure 6B), nicotine apparently did not evoke response; however, this cell also responded to capsaicin suggesting the nociceptive phenotype. On average, almost half of neurons (43%, 70/163 cells) responded to nicotine suggesting that nAChRs can have an important role in trigeminal nociception. Consistent with previous data, 34% of neurons (56/163) were sensitive to application of 200 nM capsaicin. Part of them (12/56 cells) were also sensitive to nicotine.

**Figure 7:**
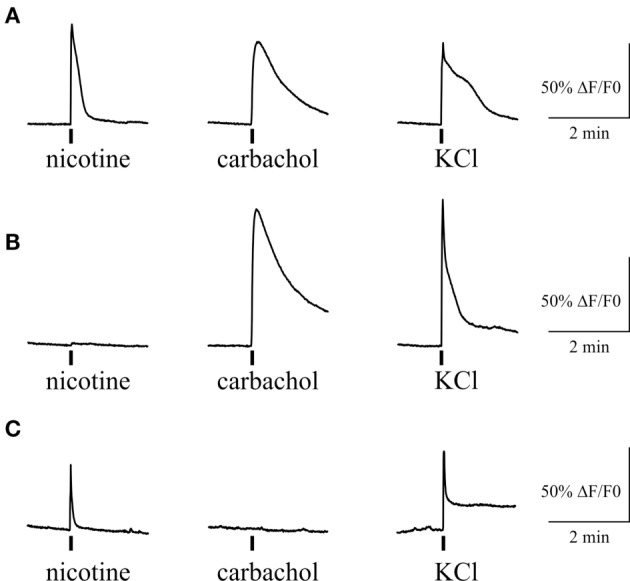
**Intracellular Ca^2+^ responses induced by nicotine and carbachol in trigeminal neurons**. **(A)** Example of calcium transients induced by 100 µM nicotine and 50 µM carbachol. **(B)** Example of calcium transient induced by application of 50 µM carbachol in nicotine-negative neuron. **(C)** Example of calcium transient induced by 100 µM nicotine in carbachol-negative neuron. All agonists were applied for 2 s with at least 2-min intervals between applications. Application of 50 mM KCl was used as a marker for identification of neurons. Statistical analysis of these experiments is given in the Section “Results.”

We also tested the action of carbachol to address the presence of mAChRs and their co-expression with nicotinic receptors in individual trigeminal neurons (Figure 7). For this purpose we first applied 100 µM nicotine with subsequent application of 50 µM carbachol. We found that 71% of neurons (82/116) responded to carbachol, whereas 70% of these cells were responsive also to nicotine application. Thus, there were neurons that responded only to carbachol application (Figure 7B) suggesting functional expression of mAChRs in trigeminal neurons.

## Discussion

In this study, we provide for a first time a detailed analysis of cholinergic regulation of nociception in meninges, which is supposed to be an origin site for migraine pain. Activation of firing in trigeminal nerve terminals was induced by the natural agonist ACh and its stable analog carbachol. The action of carbachol was prevented by atropine suggesting presence of muscarinic receptors. Similar size effect was induced by nicotine indicating also expression of nicotinic receptors in the nociceptive fibers. Our data are consistent with view that trigeminal nerve fibers represent a target for the pro-nociceptive action of ACh released from parasympathetic fibers innervating meninges. The pro-nociceptive role of endogenous ACh was detected in a CGRP-sensitized meningeal preparation. Consistent with data in meninges, isolated trigeminal neurons also responded to nicotine and carbachol by generation of Ca^2+^ transients. In contrast to nerve endings, residual mast cells did not respond to nicotine but readily degranulated their content to application of carbachol. These data provide a mechanistic interpretation for parasympathetic innervation of meninges and explain such phenomena as carbachol-induced headache in humans.

The physiologically important interaction between parasympathetic and trigeminal sensory systems in meninges has been suggested earlier (5–8). However, the fact of ACh action on trigeminal nerve terminals and molecular mechanisms underlying these processes remained unknown to date. Therefore, our primary aim was to shed a light on the molecular mechanisms of communication between parasympathetic and nociceptive trigeminal signaling. Both two main type of cholinesterases (AChE and BuChE), known to be involved in the metabolism of ACh, we found in perivascular neurofilament-rich nerve fibers around big meningeal vessels. The presence of neurofilaments (44) indicated that most of AChE- and BuChE-positive axons were myelinated. The previously obtained data (45) suggested that such nerve fibers could be parasympathetic in nature. Although a very weak enzymatic activity of AChE was also discovered in axons diffusely spread in the meningeal tissue (45), the perivascular nerve fibers are primarily implicated in ACh signaling in meninges.

Inhibition of cholinesterase activity, which is associated with increased level of endogenous ACh, intensified the nociceptive trigeminal firing in CGRP-sensitized dura mater. Thus, in experimental migraine-like conditions induced by CGRP (46), endogenous ACh is able to produce the pro-nociceptive effect, which supports the pathophysiological role of such type of signaling. This effect is mainly mediated through nAChRs, since the nicotinic agonist d-tubocurarine was able to prevent the action of neostigmine, a cholinesterase inhibitor.

Our data suggest that in dura mater, sensory afferent nerve endings are expected to be the target for the excitatory action of nicotine. This is consistent with data from other tissues, such as skin or nasal epithelium, where nicotine can directly activate nociceptive nerve endings (36, 47–50).

Somas of trigeminal neurons are the most common model of trigeminal nerve endings. Our results obtained in isolated trigeminal neurons were in agreement with data from hemiskull preparation. Indeed, we found a strong enhancement of intracellular calcium concentration in trigeminal neurons in response to nicotine in about half of trigeminal neurons. Moreover, in cultures that are lacking mast cells, essential fraction of neurons was also activated by carbachol consistent with presence of mAChRs in trigeminal afferents.

Nicotinic AChRs presented in sensory neurons have diverse subunit composition (51). All neuronal nAChR subunits were detected at mRNA level in trigeminal and dorsal root ganglion neurons, but α7, α3β4, and α6β4 nAChRs appeared to be the main functionally active receptor subtypes in sensory neurons (33, 35, 52–55). Metabotropic mAChRs are likely presented on cell bodies and nerve endings of primary afferents, and the functional expression of M2, M3 and M4 subtypes was reported earlier (40, 49, 56, 57). However, in contrast to nAChRs producing mainly the excitatory effect on sensory neurotransmission (37, 47), activation of mAChRs in primary afferents can also inhibit the nociceptive activity on the periphery (5, 40, 49) and in the spinal cord (58). The functional outcome, therefore, depends on density and mAChR subtypes expressed in trigeminal nerve endings. Thus, the role of mAChR subtypes in trigeminal neurons innervating dura mater deserves the special investigation with specific ligands for multiple types of mAChRs.

In contrast to nicotine, carbachol provoked a massive degranulation of a significant portion of meningeal mast cells. These immune cells are localized in close proximity to dural nociceptive nerve fibers (21, 59) and are able to secrete numerous pro-nociceptive mediators (21, 59–62). Degranulation of dural mast cells can lead to persistent activation of dural nociceptors (41) and the headache pain pathway (22, 63). Interestingly, according to some reports, mast cells degranulation is modulated by sex hormones in a gender-selective fashion, with mast cells from females being more susceptible than mast cells in males (64). However, we did not observe any sex-dependent difference in cholinergic activation of dural mast cell degranulation for male and female rats, probably due to young age of these animals. Nevertheless, our data suggest that in rat dura mater carbachol might indirectly activate nociceptors provoking mast cells degranulation through mAChRs.

**Figure 8:**
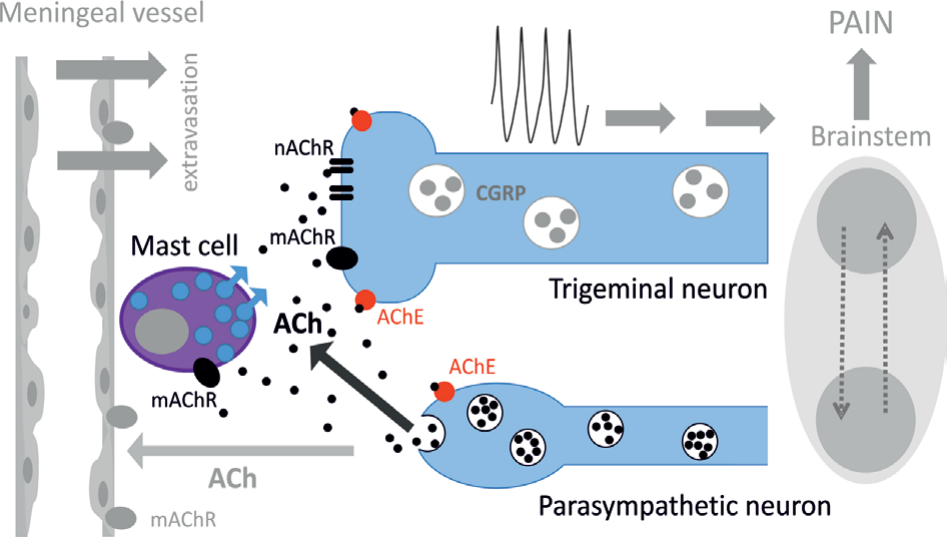
**Schematic representation of cholinergic signaling in peripheral nociception in meninges**. Meninges have a dual innervation by somatic trigeminal nerves and postganglionic parasympathetic nerve fibers. Acetylcholine (ACh) released from parasympathetic nerves can activate mast cells *via* muscarinic ACh receptor (mAChR) and trigeminal nerve endings both *via* mAChR and nicotinic ACh receptor (nAChR). This concerted activation of ACh receptors (AChRs) results in generation of the nociceptive firing in primary afferents. The lifespan of ACh is limited by activity of acetylcholinesterase (AChE), which destroys this neurotransmitter. Potentially, ACh can also approach small meningeal vessels producing vasodilation and plasma protein extravasation most likely *via* mAChRs (8). At the level of the brainstem, these two systems can interact *via* the trigemino–parasympathetic reflex leading to sustained neuronal activity. The outcome of cholinergic activation in meninges is the release of pro-inflammatory transmitters and cytokines from mast cells, extravasation, excitation, and sensitization of nociceptive afferents resulting in trigeminal pain.

Figure 8 provides a schematic presentation of results obtained in this study along with some previous key observations on the role of parasympathetic nerves in meninges. Thus, postganglionic parasympathetic nerve fibers can release ACh degranulating dural mast cells mainly *via* mAChRs. Degranulation of mast cells is associated with release of multiple pro-inflammatory transmitters and cytokines supporting neuroinflammation in meninges. Our recent study indicated the key role of serotonin derived from degranulated mast cells in activation of trigeminal nerve terminals (41). ACh can even directly activate trigeminal nerve endings both *via* mAChRs and nAChRs providing essential nociceptive firing in primary afferents. The main migraine mediator neuropeptide CGRP could be released from peptidergic nerve terminals and be involved in facilitation of cholinergic signaling. This could be either *via* enhanced ACh release, as CGRP can induce Ca^2+^ transients in certain cell types including neurons (65, 66), or *via* facilitation of ACh signaling due to CGRP-induced membrane traffic of cholinergic receptors, similar to ATP-gated P2X3 receptors (67). Thus, a complex activity of CGRP could be implicated in its modulatory action in meninges, and this issue deserves further studies.

Like in other tissues, ACh signaling depends on availability and activity of AChE and BuChE, which both present in meninges. Previous studies revealed that ACh released during stimulation of the SPG can induce vasodilation and plasma protein extravasation in dura mater *via* mAChRs (8). All these mechanisms should largely intensify the nociceptive traffic to the brainstem to promote headache and support the trigemino–parasympathetic reflex (6).

## Conclusion

The pro-nociceptive cholinergic effects observed in the current study likely involve several molecular targets such as nAChRs and mAChRs in trigeminal nociceptive neurons and mAChRs in mast cells provoking release of pro-inflammatory agents. Thus, ACh derived from parasympathetic nerve fibers could be implicated in trigeminal pain and, probably, contribute to induction of headache in conditions favoring the release of this key neurotransmitter of the autonomous nervous system.

## Ethics Statement

All experimental procedures were performed in accordance with the European Community Council Directive of September 22, 2010 (2010/63/EEC), and the experimental protocol was approved by the Animal Care and Use Committee of the University of Eastern Finland (license EKS-004-2014). All efforts were made to minimize the number of animals used and their suffering.

## Author Contributions

Design of the study: IS, EN, and RG. Conducting the study and analysis: IS, NM, PA, and LN. Manuscript preparation: IS, NM, and RG.

## Conflict of Interest Statement

The authors declare that the research was conducted in the absence of any commercial or financial relationships that could be construed as a potential conflict of interest.

## Abbreviations

PD, postnatal day; ACh, acetylcholine; mAChR, muscarinic acetylcholine receptor; nAChR, nicotinic acetylcholine receptor; AChE, acetylcholinesterase; BuChE, butyrylcholinesterase; TG, trigeminal ganglion.
